# SurfDet3D: Surface-Aware Geometric Representation and Quality-Aware 3D Detection of Small Defects on Ceramic Package Substrates

**DOI:** 10.3390/s26206547

**Published:** 2026-10-15

**Authors:** Zhuang Lv, Zhipeng Yan, Yu Yuan, Lei Zhang, Jiawei He, Weicai Lin, Zhiya Mu, Jun Wang, Furi Jin, Xin He

**Affiliations:** 1 Changchun Institute of Optics, Fine Mechanics and Physics, Chinese Academy of Sciences, Changchun 130033, China; 2 University of Chinese Academy of Sciences, Beijing 100049, China; 3 Jiangxi Provincial Department of Transportation, Nanchang 330036, China

**Keywords:** ceramic package substrate, 3D defect detection, sparse voxel network, surface geometry, localization quality estimation

## Abstract

**Highlights:**

**What are the main findings?**

- SurfDet3D combines surface-aware geometric representation with quality-aware scoring to preserve weak defect cues and improve prediction ranking in sparse point clouds.
- On CPS3D-Det at IoU = 0.5, SurfDet3D achieves 46.38% *AP*_3*D*,*R*40_ and 45.42% *AP_BEV_*_,*R*40_ with only 3.53 M parameters.

**What are the implications of the main findings?**

- Explicit surface geometry and localization-aware confidence estimation improve small-defect detection under severe foreground–background imbalance.
- SurfDet3D combines competitive detection accuracy with a compact 3.53 M-parameter architecture for ceramic package substrate inspection.

**Abstract:**

Accurate 3D detection of surface defects is essential for quality control in the manufacturing of ceramic package substrates. However, this task faces two major challenges: the reliable representation of subtle local surface geometric anomalies and the accurate localization and reliable candidate ranking of small defects with diverse morphologies. To address these challenges, we propose SurfDet3D, which preserves within-column relative geometry across multiple stages of a sparse backbone and introduces bi-scale surface statistics to enhance subtle geometric features. It further combines whole-box geometric constraints with localization quality estimation to improve bounding-box regression and candidate ranking. Experiments on the public CPS3D-Det benchmark show that SurfDet3D achieves *AP*_3*D*,*R*11_, *AP_BEV_*_,*R*11_, *AP*_3*D*,*R*40_, and *AP_BEV_*_,*R*40_ values of 48.13%, 46.45%, 46.38%, and 45.42%, respectively. These results demonstrate the effectiveness of SurfDet3D in representing subtle local surface geometric anomalies and improving the localization and candidate ranking of small defects with diverse morphologies. Ablation studies further verify the contribution of each proposed component.

## 1. Introduction

Ceramic package substrates (CPSs) combine high thermal conductivity, electrical insulation, thermal stability, and corrosion resistance and are therefore widely used in power semiconductor devices [1], aerospace electronics [2], power modules for new energy vehicles [3], and 5G/6G communication systems [4]. During manufacturing, micrometer-scale defects, including cracks, pinholes, bubbles, scratches, dents, protrusions, and stains, may form on CPS surfaces and compromise device performance and reliability. Accurate detection of these defects is therefore essential for quality control in advanced electronic packaging.

Deep learning has been widely applied to CPS defect detection using 2D images and 3D point clouds [5,6,7,8,9,10,11,12,13,14,15]. Unlike 2D methods, which primarily rely on grayscale, color, and texture information, 3D point clouds directly capture surface relief and spatial morphology. However, CPS defects often appear as subtle, low-amplitude perturbations and vary substantially in scale, orientation, aspect ratio, and boundary completeness. Their detection therefore presents two major challenges: the reliable representation of subtle local surface geometric anomalies and the accurate localization and reliable candidate ranking of small defects with diverse morphologies.

Existing methods enhance subtle-defect features through data balancing, attention mechanisms, multiscale fusion, and detail restoration [5,6,7,8,9,10,11], but 2D appearance features cannot directly characterize vertical surface variations. In voxel-based networks, voxelization and successive downsampling may further attenuate subtle geometric signals. Meanwhile, center-based 3D detectors such as AIFAD [15] generate candidates from center heatmaps and regress the remaining box parameters through separate branches. Candidate ranking is therefore primarily determined by center-heatmap confidence, which does not explicitly reflect the localization quality of the complete predicted box. These limitations motivate the explicit modeling of surface geometry, the introduction of whole-box geometric constraints, and the estimation of localization quality.

To address these challenges, we propose SurfDet3D, a single-stage 3D detector that integrates surface-aware feature enhancement with quality-aware sparse detection. Local relative geometry modulation (LRG) derives top-relative elevation and within-column occupancy variation from sparse voxel columns and uses them to modulate active voxel features at multiple backbone stages. Bi-scale surface guidance (BSSG) extracts surface statistics at two bird’s-eye-view (BEV) scales and recalibrates the BEV representation through channel and spatial gates. Joint geometric constraints (JGCs) combine axis-aligned 3D distance-IoU (DIoU) with orientation-aware BEV envelope DIoU to regularize whole-box geometry. In addition, a localization quality branch (QB), supervised by rotated 3D intersection over union (IoU), calibrates candidate ranking according to predicted localization quality.

Our contributions are summarized as follows:

1. We propose SurfDet3D, a unified single-stage 3D detector designed for small surface defects on ceramic package substrates. It integrates surface-aware feature representation with geometry- and quality-aware detection in a sparse voxel framework, providing an end-to-end solution for subtle-defect representation, bounding-box localization, and candidate ranking.
2. We introduce LRG and BSSG to strengthen the representation of subtle local surface geometric anomalies. LRG preserves within-column relative geometry across multiple stages of the sparse backbone, whereas BSSG provides bi-scale cross-column surface context. Together, they retain subtle vertical geometric cues and capture defect morphology over different spatial extents.
3. We design JGC and QB to address the second challenge: accurately localizing and reliably ranking small defects with diverse morphologies. JGC regularizes whole-box geometry in both 3D space and the BEV plane, while QB incorporates predicted localization quality into candidate scoring, thereby improving geometric consistency and the alignment between classification confidence and localization quality.
4. SurfDet3D employs a compact sparse voxel architecture and a single-stage inference pipeline with 3.527 M parameters, providing a favorable balance between detection accuracy and model size.

Experiments on the public CPS3D-Det benchmark under the AIFAD validation protocol demonstrate the effectiveness of the proposed components and the overall performance of SurfDet3D.

## 2. Related Work

### 2.1. Representation of Subtle Local Surface Geometric Anomalies

Research on the representation of subtle CPS defects has primarily focused on data balancing, discriminative feature enhancement, detail restoration, and generalization under limited-data conditions. Huang et al. [5] combined K-means analysis, enhanced filtering, and balanced sampling to alleviate class imbalance. Liao et al. [6] integrated GhostNet and CBAM into GhostC-YOLOX to enhance defect-relevant channel and spatial features. Xin et al. [7] used dilated depthwise separable convolutions and shallow-to-deep fusion to improve contextual and boundary representations, while He et al. [8] strengthened discriminative feature extraction and cross-level semantic interaction through PFEI-Net. For limited-sample and unseen-class settings, DCCNet [9] employed cross-set attention and tail enhancement, EDNet [10] combined diffusion-based reconstruction with cross-modal fusion, and HsINet [11] jointly modeled spatial and channel relationships while preserving high-frequency details.

Although these methods improve the representation of texture, edges, and other appearance cues in 2D images, they cannot directly characterize vertical variations relative to the substrate surface. Voxel-based 3D networks can learn geometric relationships from point coordinates and neighboring features; however, micrometer-scale perturbations on nearly planar surfaces remain susceptible to height variation, voxel quantization, and feature downsampling. Existing CPS defect detectors therefore lack an explicit relative geometric representation defined with respect to local voxel occupancy and preserved across multiple backbone stages.

### 2.2. 3D Localization and Quality-Aware Ranking in Existing Defect Detectors

Existing studies have progressively improved CPS defect localization through discriminative feature extraction, multiscale modeling, and geometric representation. Huang et al. [12] combined deep classification networks with YOLOv3 to perform defect recognition and spatial localization. Wang et al. [13] used partial convolutions, hierarchical attention, and adaptive loss weighting to improve multiscale localization under class imbalance. Guo et al. [14] introduced structural refinement and quality assessment into point-cloud segmentation, while Yu et al. [15] developed CPS3D-Det and AIFAD, which aggregate multiscale sparse voxel features and directly regress 3D bounding boxes from center-heatmap responses.

General-purpose point-cloud detectors provide important methodological foundations for 3D localization. Part-A^2^ [16] and PV-RCNN [17] improve proposal refinement through intra-object part aggregation and point–voxel feature abstraction, respectively. PointPillars [18] encodes points within vertical columns for efficient BEV processing, while CenterPoint [19] represents objects by their centers and regresses the remaining box attributes, establishing an efficient center-based detection paradigm. PillarNet [20] strengthens pillar feature learning and spatial–semantic fusion, SECOND [21] improves voxel-based feature extraction efficiency through sparse convolution, and VoxelNeXt [22] further enables object prediction directly from fully sparse voxel features.

Several methods have also incorporated localization quality into bounding-box regression and candidate scoring. Part-A^2^ [16] re-scores and refines proposals using aggregated part geometry, PV-RCNN [17] employs IoU-guided confidence prediction, and PillarNet [20] combines orientation-decoupled IoU regression with an IoU-aware prediction branch. These approaches demonstrate that classification confidence alone may not reliably reflect the localization quality of a complete predicted box. However, proposal refinement generally introduces additional computation, whereas existing center-based CPS detectors such as AIFAD [15] still rely primarily on center-heatmap confidence for candidate ranking. Moreover, current CPS methods do not explicitly exploit multiscale surface statistics, impose direct geometric constraints on complete predicted boxes, or incorporate predicted localization quality into candidate ranking. Accordingly, SurfDet3D uses BSSG to complement the within-column relative geometry modeled by LRG with bi-scale cross-column surface context, while JGC and QB improve whole-box localization and localization-quality-aware candidate ranking, respectively.

## 3. Surface-Aware 3D Detection of Small Surface Defects

### 3.1. Overall Framework

**Figure 1:**
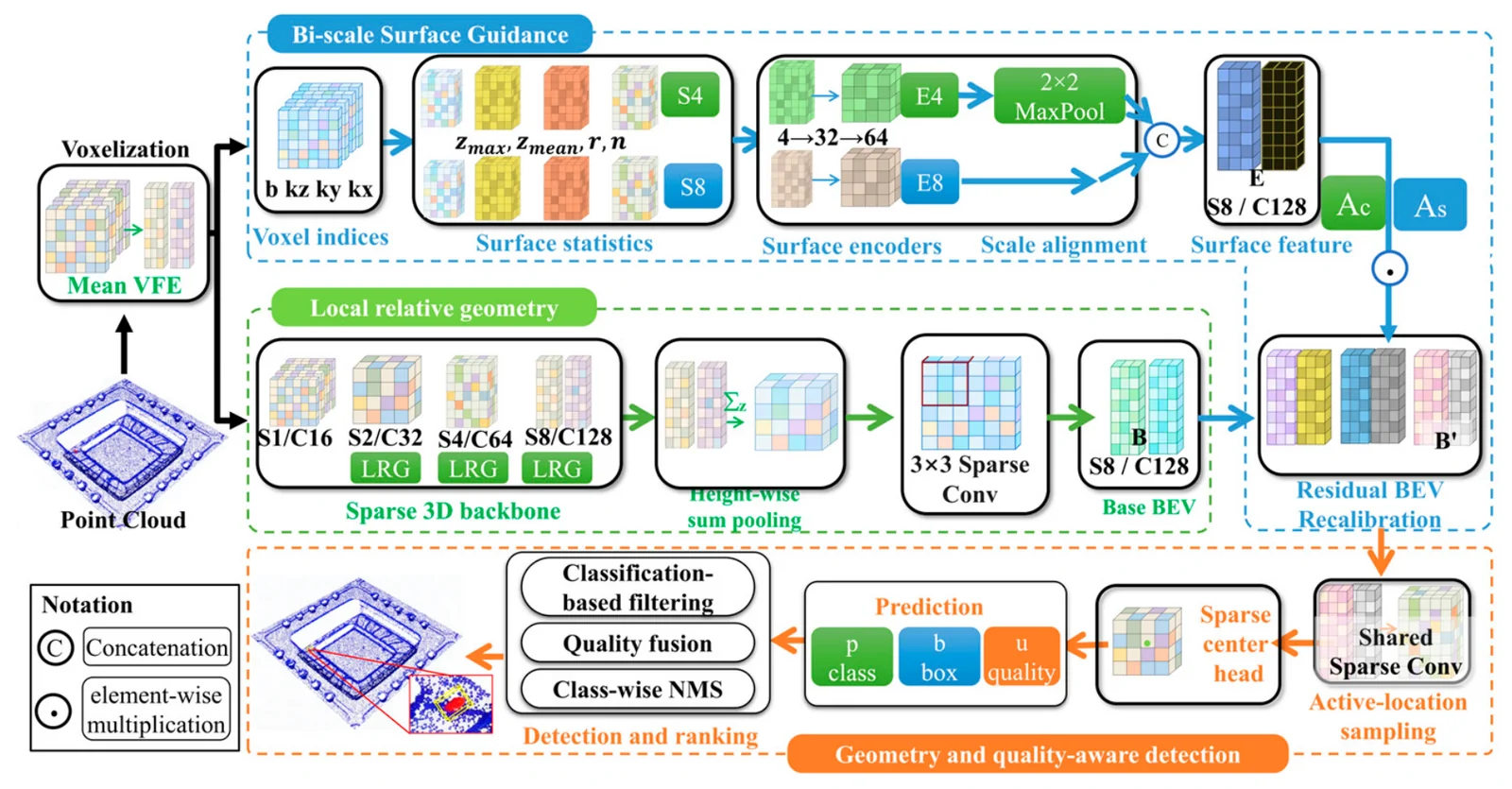
Overall architecture of SurfDet3D. Mean VFE denotes mean voxel feature encoding; S and C indicate spatial stride and channel count, respectively.

The overall framework of SurfDet3D is shown in Figure 1. The input point cloud is first voxelized into a sparse 3D tensor and processed by a four-stage sparse backbone. To reliably represent subtle local surface geometric anomalies, LRG derives relative within-column occupancy descriptors and uses them to modulate voxel features at multiple backbone stages, thereby preserving weak vertical variations throughout hierarchical feature extraction. The deepest sparse features are then aggregated along the height dimension to form the base BEV representation. In parallel, BSSG derives surface statistics from the active input voxels, encodes them at two BEV scales, and generates channel and spatial gates to recalibrate the base BEV features. Together, LRG and BSSG provide complementary within-column relative geometry and cross-column surface context for subtle defects with different spatial extents.

The recalibrated BEV features are sampled at active locations and passed to a sparse center-based detection head. To improve the localization and candidate ranking of small defects with diverse morphologies, JGC combines axis-aligned 3D DIoU with orientation-aware BEV envelope DIoU to constrain the center, dimensions, vertical overlap, and orientation-related geometry of complete predicted boxes. QB further estimates localization quality under rotated 3D IoU supervision and fuses it with classification confidence to improve candidate ranking before non-maximum suppression (NMS). All components are jointly optimized within a single-stage, end-to-end detection framework.

### 3.2. Cross-Stage Representation of Subtle Local Surface Geometric Anomalies

**Figure 2:**
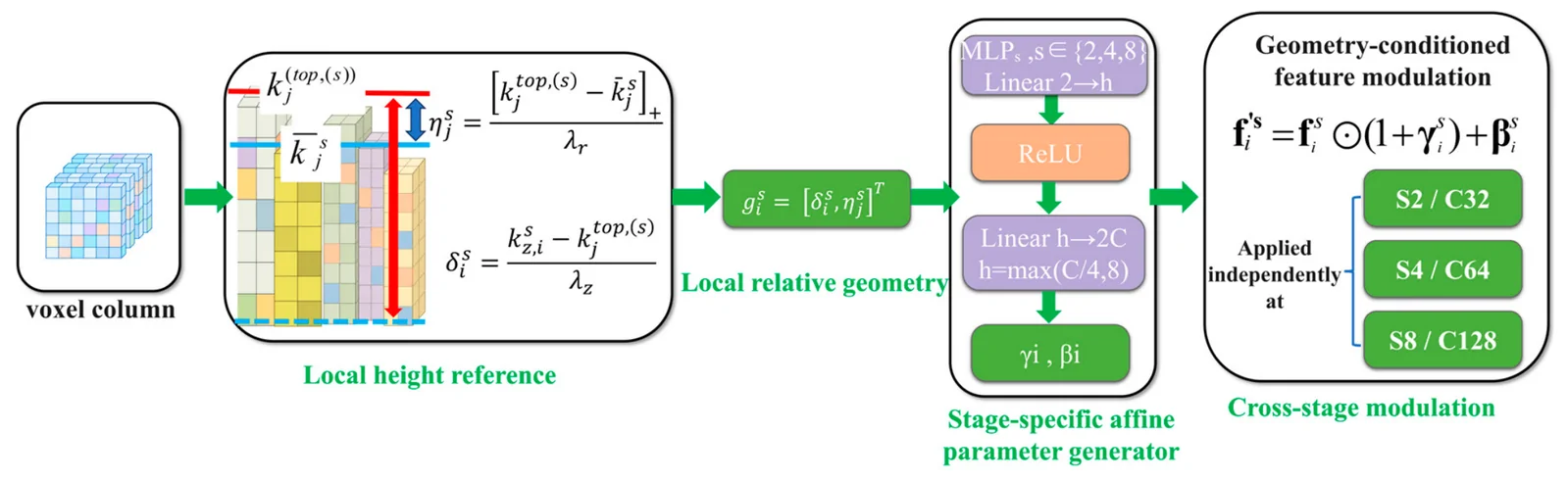
Local relative geometry and feature modulation at multiple backbone stages. MLP denotes a multilayer perceptron; γ and β denote the multiplicative and additive modulation terms, respectively.

Dents, protrusions, and shallow cracks on CPSs often appear as low-amplitude vertical perturbations. Representations learned solely from absolute coordinates may be affected by variations in workpiece height, local warpage, and successive downsampling. As illustrated in Figure 2, LRG therefore establishes a local height reference within each active voxel column and maps the resulting relative geometry to voxel-wise, channel-wise affine parameters at multiple backbone stages. This design preserves subtle vertical occupancy information throughout hierarchical feature extraction.

#### 3.2.1. Local Relative Geometry Within Voxel Columns

At backbone stride *s* ∈ {2,4,8}, let $\Omega_{j}^{s}$ denote the set of active voxels in BEV projection cell *j*, and let $k_{z,i}^{s}$ be the vertical index of voxel *i*. The highest and mean occupied layers are defined as:

(1) $$k_{j}^{\mathrm{top},(s)}=\underset{i\in \Omega_{j}^{s}}{\max}\;k_{z,i}^{s},\;{\bar{k}}_{j}^{s}=\frac{1}{\left|\Omega_{j}^{s}\right|}\sum_{i\in \Omega_{j}^{s}}k_{z,i}^{s}$$

The highest occupied layer defines the upper occupancy boundary of the voxel column, whereas the mean occupied layer characterizes its overall vertical distribution. Both quantities are derived from discrete voxel occupancy rather than the continuous heights of the original point cloud. The relative elevation of voxel *i* and the occupancy variation of column *j* are defined as:

(2) $$\delta_{i}^{s}=\frac{k_{z,i}^{s}-k_{j}^{\mathrm{top},(s)}}{\lambda_{z}},\;\eta_{j}^{s}=\frac{{\left[k_{j}^{\mathrm{top},(s)}-{\bar{k}}_{j}^{s}\right]}_{+}}{\lambda_{r}}$$

where [·]_+_ denotes *max*(·, 0). The constants *λ_z_* = 10 and *λ_r_* = 5 are fixed, empirically chosen normalization factors for voxel-index differences. Since $k_{z,i}^{s}\leq k_{j}^{\mathrm{top},(s)}$, the relative elevation $\delta_{i}^{s}$ is non-positive and encodes the signed vertical offset of voxel *i* relative to the top of its column. The column-wise term $\eta_{j}^{s}\geq 0$ represents the vertical dispersion of occupied layers and is shared by all active voxels in the same projection cell. The local geometry descriptor is:

(3) $$g_{i}^{s}=\;{\left[\delta_{i}^{s},\eta_{j}^{s}\right]}^{T}$$

Both components are zero when a projection cell contains only one occupied layer. Since $g_{i}^{s}$ depends on relative occupancy, it is invariant to a common additive shift of all vertical indices within the column, thereby reducing its sensitivity to overall workpiece-height offsets. BSSG complements this within-column relative geometry by modeling surface context across neighboring columns.

#### 3.2.2. Cross-Stage Local Relative Geometry Modulation

To prevent relative geometric cues from being attenuated during feature propagation, LRG applies independently parameterized modulation units at strides 2, 4, and 8, corresponding to 32, 64, and 128 feature channels, respectively. At each stride, a two-layer perceptron maps the local geometry descriptor to channel-wise scaling and shifting parameters:

(4) $$\left[\gamma_{i}^{s};\beta_{i}^{s}\right]=W_{2}^{\left(s\right)}\mathrm{ReLU}\left(W_{1}^{\left(s\right)}g_{i}^{s}+b_{1}^{\left(s\right)}\right)+b_{2}^{\left(s\right)}$$

The hidden dimension is *max*(*C*/4, 8), and the output dimension is 2C, where C denotes the number of channels at the current stage. The active voxel feature is updated by:

(5) $$f_{i}^{\prime \left(s\right)}=\;f_{i}^{s}⊙\left[1+\gamma_{i}^{s}\right]+\beta_{i}^{s}$$

Here, ⊙ denotes channel-wise, element-wise multiplication. The geometry-conditioned scale and shift adjust the active voxel features, while the identity term retains the original feature pathway. LRG links an explicit column-relative occupancy reference to hierarchical feature learning: independently parameterized modulation units adapt this conditioning to the spatial resolution and receptive field of each stage. The sparse coordinates, active voxel set, and tensor dimensions remain unchanged, allowing relative occupancy geometry to participate directly in feature propagation.

### 3.3. Surface-Guided Localization and Quality-Aware Candidate Ranking

CPS defects vary substantially in scale, orientation, spatial extent, and boundary completeness. Compact defects require sensitivity to fine local relief, whereas elongated scratches and cracks require a broader surface context. In addition, center-heatmap confidence indicates the likelihood of a defect center but does not directly reflect the localization accuracy of the complete predicted box. BSSG therefore incorporates bi-scale cross-column surface context, JGC imposes whole-box geometric constraints, and QB estimates localization quality to improve candidate ranking.

#### 3.3.1. Bi-Scale Surface Guidance for Residual BEV Recalibration

**Figure 3:**
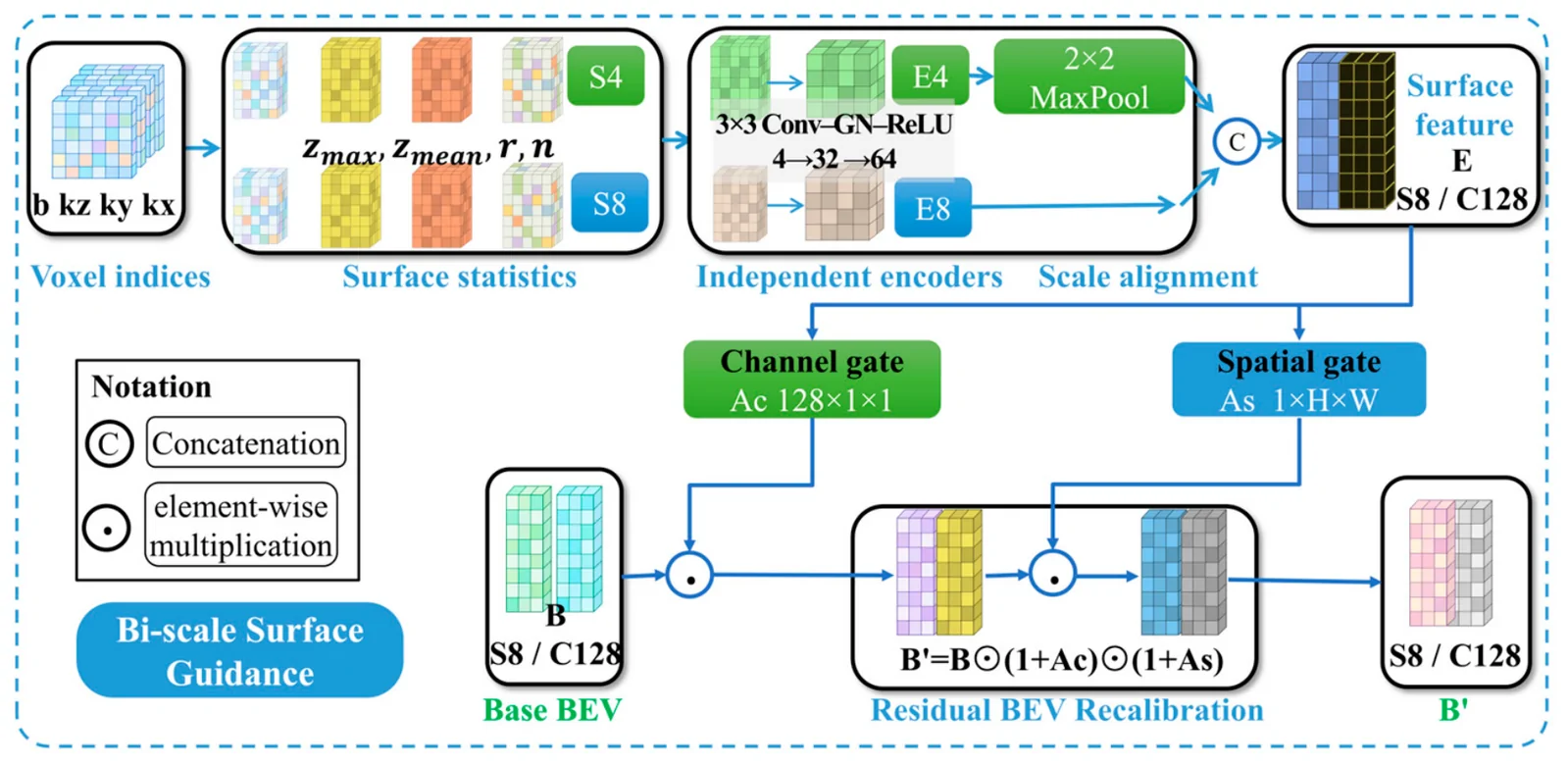
Bi-scale surface guidance and residual BEV recalibration.

BSSG constructs surface descriptors directly from the active input voxels on stride-4 and stride-8 BEV grids, as illustrated in Figure 3. The finer grid preserves local surface relief, whereas the coarser grid captures broader surface structures. For *s* ∈ {4, 8}, let $V_{j}^{s}$ denote the active voxels in projection cell *j*. The occupied-layer height proxy of voxel *i* is defined as:

*z_i_* = (*k_z_*_,*i*_ − 1)*d_z_* + *z_min_* (6)

Here, *k_z_*_,*i*_ is the discrete vertical voxel index, *d_z_* is the voxel size, and *z_min_* is the lower bound of the valid point-cloud range. The value *z_i_* is a network-input proxy obtained through a fixed affine mapping of the discrete index rather than the exact physical height of the voxel center. Its fixed origin introduces a constant offset into the maximum and mean components but does not alter relative variations within the same projection cell.

For each nonempty cell, the maximum and mean occupied-layer height proxies are:

(7) $$z_{j}^{\max,s}=\underset{i\in V_{j}^{s}}{\max}\;z_{i},\;z_{j}^{\mathrm{mean},s}=\frac{1}{\left|V_{j}^{s}\right|}\sum_{i\in V_{j}^{s}}z_{i}$$

The corresponding vertical variation and number of occupied voxels are defined as follows:

(8) $$r_{j}^{s}=\;{\left[z_{j}^{\max,s}-z_{j}^{\mathrm{mean},s}\right]}_{+},\;n_{j}^{s}=\;\left|V_{j}^{s}\right|$$

Here, $\;n_{j}^{s}$ represents the number of nonempty 3D voxels rather than the number of raw points. Empty cells are assigned zero-valued descriptors. To reduce differences in units and dynamic ranges, the four statistics are normalized and concatenated as follows:

(9) $$h_{j}^{s}=\;{\left[\frac{z_{j}^{\max,s}-z_{\min}}{z_{\max}-z_{\min}},\frac{z_{j}^{\mathrm{mean},s}-z_{\min}}{z_{\max}-z_{\min}},\tanh\left(\frac{r_{j}^{s}}{2d_{z}+\varepsilon }\right),\frac{\log\left(1+n_{j}^{s}\right)}{10}\right]}^{T}$$

Here, *z*_max_ is the upper bound of the valid point-cloud range, and the added constant ensures numerical stability.

The stride-4 and stride-8 surface maps are processed by independent encoders, each comprising two 3 × 3 convolution–group normalization–ReLU blocks with channel dimensions 4 → 32 → 64. A 2 × 2 max-pooling operation aligns the stride-4 features with the stride-8 features before concatenation:

(10) $$E\;=\;\mathrm{Concat}\left[\mathrm{MaxPoo}l_{\left(2\times 2\right)}\left(E_{4}\right),E_{8}\right],E\in R^{B\times 128\times H_{8}\times W_{8}}$$

Let $B\in R^{B\times 128\times H_{8}\times W_{8}}$ denote the base BEV features produced by the main stream. The fused surface representation *E* is fed into channel and spatial gating branches. The channel branch uses global average pooling to model channel dependencies, while the spatial branch identifies BEV regions with distinctive surface patterns. The resulting channel gate *A_c_* ∈ (0,1)*^B^*^×128×1×1^ and spatial gate $A_{s}\in {\left(0,1\right)}^{\left(B\times 1\times H_{8}\times W_{8}\right)}$ recalibrate the base features as:

*B*^′^ = *B*⊙(1 + *A_c_*)⊙(1 + *A_s_*) (11)

Both gates use sigmoid activation, and the residual formulation retains the base BEV response while introducing surface-conditioned feature enhancement. Channel and spatial recalibration follow the established attention principle [23], with the guidance here supplied by explicit surface statistics encoded at two BEV scales. This separate geometry pathway connects local occupied-layer variation and broader cross-column context to the backbone features. The recalibrated map is sampled at the original active BEV coordinates to produce the sparse representation used by the detection head.

#### 3.3.2. Joint Geometric Constraints and Quality-Aware Sparse Detection

**Figure 4:**
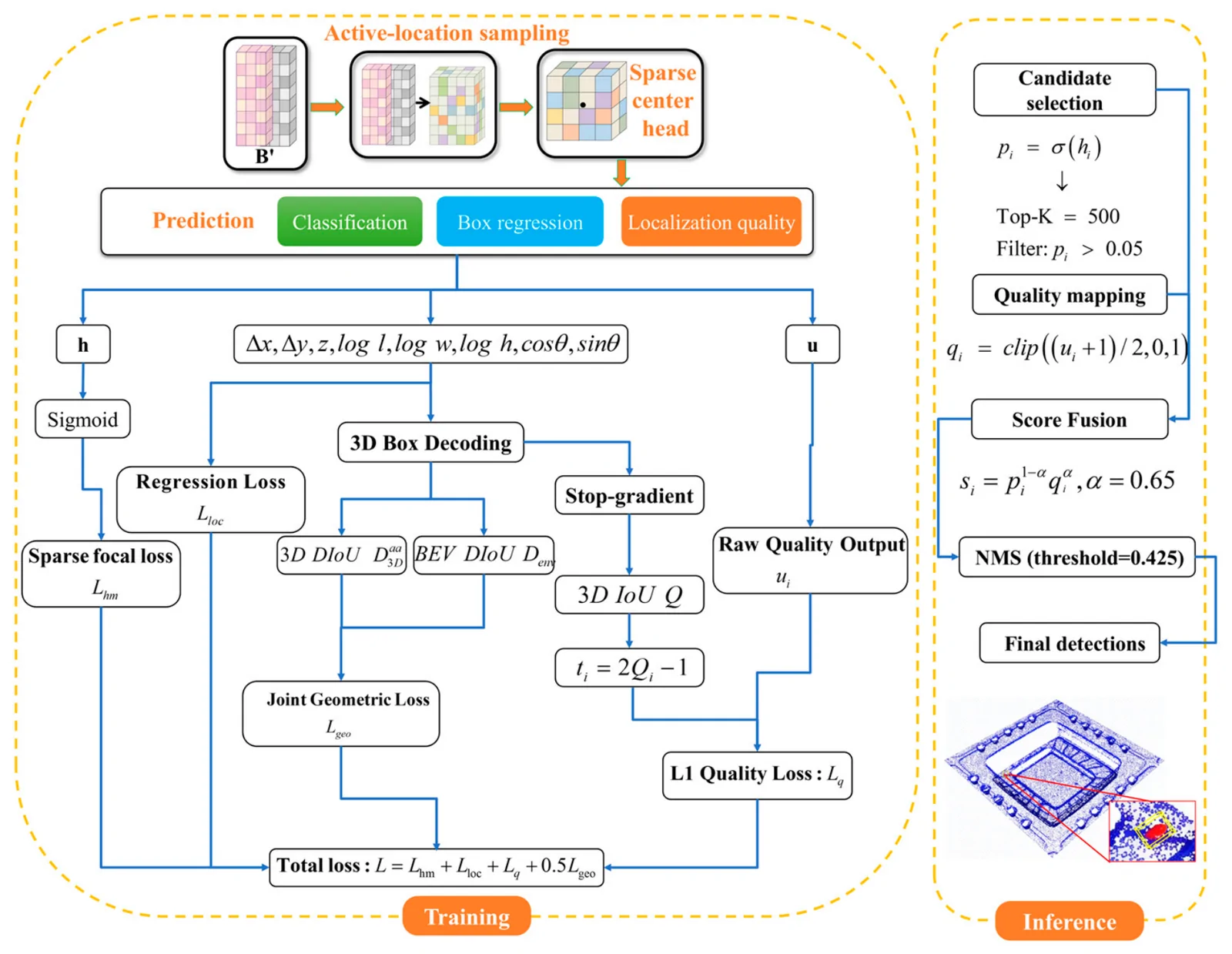
Training and inference workflow of the joint geometric-constraint and quality-aware sparse detection head.

As shown in Figure 4, the enhanced sparse BEV features are processed by a shared 2D submanifold convolution and a center-based sparse detection head. At each active BEV location, the head predicts a class heatmap *h*, planar center offsets (Δ*x*,Δ*y*), absolute height *z*, log-dimensions (*log l*, *log w*, *log h*), orientation encoding (*cos θ*, *sin θ*), and a raw localization quality value *u*.

During training, each continuous ground-truth center is mapped to the stride-8 BEV coordinate system, and its nearest active location is selected for box regression. The heatmap target is obtained by taking the element-wise maximum of a continuous Gaussian response centered at the ground-truth center and a discrete Gaussian response centered at the selected active location. Responses from multiple ground-truth boxes are also combined by element-wise maximization. The continuous response preserves the spatial relationship between the ground-truth center and the active locations, whereas the discrete response assigns a peak target of 1 to the selected regression location. The center-offset branch regresses the displacement from this active location to the continuous ground-truth center.

The class heatmap is supervised by sparse focal loss *L*_hm_. Box parameters are optimized using weighted L1 regression, with a weight of 2 for absolute height and 1 for each remaining regression term. To connect parameter-wise regression with whole-box geometry, JGC builds on the overlap and normalized center-distance formulation of DIoU [24] and combines axis-aligned 3D DIoU with yaw-dependent BEV-envelope DIoU. These terms provide complementary volumetric and planar supervision.

For a predicted box $\hat{b}$ and its ground-truth box b^*^, axis-aligned 3D DIoU is defined as

(12) $${\mathrm{DIoU}}_{3D}^{\mathrm{aa}}\left(\hat{b},b^{*}\right)={\mathrm{IoU}}_{3D}^{\mathrm{aa}}\left(\hat{b},b^{*}\right)-\frac{{\left|\left|\hat{c}-c^{*}\right|\right|}_{2}^{2}}{d_{C,3D}^{2}}$$

where $\hat{c}$ and c^*^ are the predicted and ground-truth centers, and *d_C_*_,3*D*_ is the diagonal length of their smallest enclosing axis-aligned 3D cuboid. This term constrains the box centers, dimensions, and volumetric overlap without incorporating yaw.

To introduce orientation-sensitive geometric gradients, an axis-aligned BEV envelope is constructed from the planar dimensions *l*, *w* and yaw *θ*. Its half-extents are:

(13) $$a_{x}=\frac{1}{2}\left[l\sqrt{\cos^{2}\theta +\varepsilon }+w\sqrt{\sin^{2}\theta +\varepsilon }\right],a_{y}=\frac{1}{2}\left[l\sqrt{\sin^{2}\theta +\varepsilon }+w\sqrt{\cos^{2}\theta +\varepsilon }\right]$$

The BEV envelope DIoU is:

(14) $${\mathrm{DIoU}}_{\mathrm{env}}\left(\hat{b},b^{*}\right)={\mathrm{IoU}}_{\mathrm{env}}\left(\hat{b},b^{*}\right)-\frac{{\left|\left|{\hat{c}}_{\mathrm{xy}}-c_{\mathrm{xy}}^{*}\right|\right|}_{2}^{2}}{d_{C,\mathrm{BEV}}^{2}}$$

Here, *d_C_*_,*BEV*_ is the diagonal length of the smallest rectangle enclosing both BEV envelopes. The BEV-envelope term couples planar dimensions and yaw through standard tensor operations, while explicit sine–cosine regression provides direct orientation supervision.

The two DIoU terms are equally weighted and averaged over *P* positive regression locations:

(15) $$L_{\mathrm{geo}}=\frac{1}{2\left(P+\varepsilon \right)}\overset{P}{\underset{i=1}{\sum }}[(1-{\mathrm{DIoU}}_{3D,i}^{\mathrm{aa}})+(1-{\mathrm{DIoU}}_{\mathrm{env},i})]$$

The axis-aligned 3D DIoU constrains box centers, dimensions, and vertical overlap, while the BEV-envelope DIoU complements it by constraining planar centers and extents.

Following the localization quality prediction principle in [17,20], QB estimates the rotated 3D IoU between each regressed box and its assigned ground-truth box, linking candidate confidence to whole-box localization quality.

(16) $$Q_{i}={\mathrm{IoU}}_{3D}^{\mathrm{rot}}\left(\mathrm{stopgrad}\left({\hat{b}}_{i}\right),b_{i}^{*}\right)$$

*t_i_* = 2*Q_i_* − 1 (17)

Rotated 3D IoU combines the intersection area of the rotated BEV boxes with their vertical overlap. The *stopgrad*(·) operation prevents gradients from propagating through the predicted box along the quality-target path. A linear layer produces the raw quality output *u_i_* without a final tanh or sigmoid activation. QB is trained using an L1 loss on the transformed quality target:

(18) $$L_{q}=\frac{1}{P+\varepsilon }\sum_{i=1}^{P}\left|u_{i}-t_{i}\right|$$

During inference, the raw output is mapped to $q_{i}={\mathrm{clip}}_{\left[0,1\right]}\left(\frac{u_{i}+1}{2}\right)$ and fused with class probability *p_i_*:

(19) $$s_{i}=p_{i}^{1-\alpha }q_{i}^{\alpha },\alpha =0.65$$

Top-K selection and classification-threshold filtering first define the retained candidate set. Quality fusion then adjusts candidate ordering before class-wise NMS. Supervision at positive regression locations associates QB with the localization reliability of regressed boxes, while the classification branch determines foreground confidence. This division connects geometric regression quality to final candidate ranking within the single-stage sparse detection head.

The complete training objective is defined as follows:

*L* = *L_hm_* + *L_loc_* + *L_q_* + 0.5*L_geo_* (20)

The four objectives are jointly optimized within a unified multitask framework. LRG and BSSG receive gradients through the sparse backbone and BEV feature pathways; the classification branch is supervised by *L_hm_*, the box regression branch by *L_loc_* and *L_geo_*, and QB by *L_q_*. This formulation enables end-to-end optimization of surface-aware geometric representation, whole-box regression, and localization-quality-aware candidate ranking.

## 4. Experiments and Analysis

### 4.1. Experimental Settings

#### 4.1.1. Dataset and Preprocessing

Experiments were conducted on the public CPS3D-Det dataset [15], which contains 1640 industrial point-cloud samples with a spatial resolution of 0.0025 mm and a measurement accuracy of 0.0003 mm. Raw point clouds contain approximately 119,000–812,000 points, while the provided geometry-sampled inputs retain approximately 25,000–182,000 points per sample. All surface anomalies belong to a single defect class, with morphological diversity referring to variations in scale, orientation, and spatial extent. Defective points typically account for less than 5% of each sample, resulting in pronounced foreground–background imbalance.

Following the benchmark split, 928 defective samples were used for training and 312 for validation. The remaining 170 normal samples and 230 challenging defective samples were excluded from training and checkpoint selection; the latter were used only for the qualitative analysis in Section 4.5. The defect class was mapped to Car solely for compatibility with the benchmark evaluator.

Following the benchmark preprocessing convention, point coordinates, box centers and dimensions, spatial ranges, and voxel sizes were consistently scaled by 100, preserving the physical voxel partition and geometric IoU. Box regression targets were encoded from the scaled coordinates as described in Section 3.3.2. The spatial ranges were [0, 776], [−368, 368], and [−120, 40] along the *x*, *y*, and *z*-axes, respectively. The voxel size was (0.5, 0.5, 4), corresponding to (0.005, 0.005, 0.04) mm before scaling. This grid defines sparse indexing rather than measurement resolution: MeanVFE encodes mean continuous point coordinates, while LRG and BSSG describe discrete occupancy geometry. At most five points were retained per voxel and 160,000 voxels per sample. Training augmentation included ground-truth instance sampling, random flipping along the *x*-axis, rotation within [−*π*/4, *π*/4], and scaling within [0.95, 1.05]. No random augmentation was applied during validation.

#### 4.1.2. Implementation Details and Training Strategy

Experiments were conducted on a single NVIDIA GeForce RTX 3080 Ti GPU (NVIDIA Corporation, Santa Clara, CA, USA) using PyTorch (v2.8.0) with CUDA (v12.9), with a training batch size of 4. SurfDet3D was trained end-to-end from random initialization for 500 epochs using the Adam optimizer and a cosine-annealing learning-rate schedule. The epoch count, batch size, optimizer, learning-rate settings, and data augmentation were shared with AIFAD* and VoxelNeXt*. The initial learning rate was 1 × 10^−3^, the weight-decay coefficient was 0.01, and the gradient-norm clipping threshold was 10.

The classification, box regression, and localization quality losses were each assigned a weight of 1, while the joint geometric loss was assigned a weight of 0.5. The box regression weights were set to [1,1,2,1,1,1,1,1], corresponding to the two planar center offsets, absolute height, three box dimensions, and two-dimensional orientation encoding.

During inference, classification-based Top-K selection retained at most 500 candidates per sample with a threshold of 0.05. Their scores were fused according to Equation (19) with a coefficient of 0.65, followed by class-wise rotated NMS at a threshold of 0.425. The NMS threshold was selected by maximizing the mean validation AP across the four metrics with the checkpoint and other inference settings fixed.

#### 4.1.3. Evaluation Metrics and Experimental Protocol

Detection performance was evaluated using 3D and BEV average precision under R11 and R40 recall sampling, with all AP values reported as percentages. The main comparisons and ablations used an IoU threshold of 0.5, and SurfDet3D was additionally evaluated at 0.7.

The official evaluator and evaluation settings in [15] were retained. Validation was performed every five epochs, and one checkpoint per run was selected according to validation *AP*_3*D*,*R*40_. The checkpoint at epoch 345 from the original run was retained for the complete-model results in Table 1 and Table 2. An additional 500-epoch training run was performed from random initialization using seed 688, with the data split, training configuration, and inference settings unchanged. Its results are reported in Section 4.4.1.

### 4.2. Comparison with Existing Methods

SurfDet3D was compared with the published CPS3D-Det benchmark methods and two locally evaluated baselines. Table 1 distinguishes published benchmark results from local experiments. The published results were taken from [15]. AIFAD* was trained from random initialization using the public model configuration and the shared local optimization settings in Section 4.1.2, while VoxelNeXt* used its best validated local configuration. Published results provide the primary benchmark comparison, and local results provide supplementary comparisons under the same experimental environment.

At an IoU threshold of 0.5, SurfDet3D achieved *AP*_3*D*,*R*11_, *AP_BEV_*_,*R*11_, *AP*_3*D*,*R*40_, and *AP_BEV_*_,*R*40_ values of 48.13%, 46.45%, 46.38%, and 45.42%, respectively, outperforming all other methods listed in Table 1. Compared with the published AIFAD results, the four metrics improved by 5.73, 5.32, 6.26, and 5.88 percentage points, respectively. Compared with the published VoxelNeXt results, the corresponding improvements were 7.48, 8.98, 8.61, and 10.48 percentage points. The improvements observed under both R11 and R40 indicate that the performance gain is consistent across the two AP calculation settings. These results support the overall detection performance of SurfDet3D under the public CPS3D-Det validation protocol.

At an IoU threshold of 0.7, SurfDet3D obtained *AP*_3*D*,*R*11_, *AP_BEV_*_,*R*11_, *AP*_3*D*,*R*40_, and *AP_BEV_*_,*R*40_ values of 31.84%, 18.68%, 27.70%, and 12.61%, respectively. Stricter overlap requirements increase sensitivity to planar center, dimension, and yaw errors, particularly for small and elongated boxes.

The local VoxelNeXt* results were 2.13, 3.71, 5.43, and 5.81 percentage points lower than the corresponding published values, while the AIFAD* results were lower by 2.15, 1.57, 2.01, and 1.91 percentage points. These differences may reflect variations in software dependencies, random initialization, and sparse-convolution implementations. The published values were therefore retained as the primary comparison targets, while the local results are reported as supplementary references obtained in the present experimental environment.

SurfDet3D contains 3.527 M parameters, corresponding to reductions of 0.661 M (15.8%) and 3.926 M (52.7%) relative to AIFAD* and VoxelNeXt*, respectively. Together with the highest reported values for the four AP metrics in the comparison table, this parameter count demonstrates the accuracy–model-size trade-off achieved by the proposed architecture under the stated benchmark protocol. Under the hardware and software configuration specified above, the measured single-sample inference latency of SurfDet3D was 54.31 ms. On the local platform, SurfDet3D improved *AP*_3*D*,*R*40_ by 8.27 and 14.04 percentage points over AIFAD* and VoxelNeXt*, with additional inference latencies of 15.65 and 20.06 ms, respectively.

### 4.3. Ablation Studies

Table 2 evaluates the four proposed components using a leave-one-component-out strategy under the experimental protocol described in Section 4.1. The Full model includes LRG, BSSG, JGC, and QB, whereas the Baseline disables all four components. The Baseline is an internal configuration of SurfDet3D and does not correspond to either AIFAD or VoxelNeXt in Table 1.

Removing LRG reduced the four AP metrics by 4.88, 3.80, 7.25, and 3.50 percentage points, respectively, supporting the contribution of cross-stage within-column relative geometry to the preservation of subtle vertical structures. Removing BSSG decreased the four metrics by 4.20, 7.19, 6.92, and 7.34 percentage points, respectively, with both BEV metrics decreasing by more than 7 percentage points. These results indicate that LRG and BSSG provide complementary within-column relative geometry and cross-column surface context for representing subtle local surface geometric anomalies.

Removing JGC reduced the four metrics by 6.47, 5.88, 5.40, and 8.48 percentage points, respectively, supporting the contribution of the joint 3D and BEV geometric constraints to whole-box localization. Removing QB resulted in decreases of 9.50, 6.15, 9.59, and 9.90 percentage points. Three of the four metrics exhibited their largest reductions among the leave-one-component-out configurations, indicating the importance of localization quality estimation and quality-aware score fusion for candidate ranking.

Compared with the Baseline, the Full model improved the four AP metrics by 11.31, 11.54, 11.10, and 13.54 percentage points, respectively. Overall, LRG and BSSG strengthen the representation of subtle local surface geometric anomalies, whereas JGC and QB improve the localization and candidate ranking of small defects with diverse morphologies. Their complementary contributions support the overall performance of SurfDet3D.

LRG, BSSG, and QB add 11,368, 84,897, and 16,897 trainable parameters, respectively, totaling 0.113 M parameters, or 3.21% of the complete model. JGC is evaluated only during training and adds no trainable parameters or inference operations.

### 4.4. Visualization Analysis

#### 4.4.1. Training Dynamics and Detection Results

**Figure 5:**
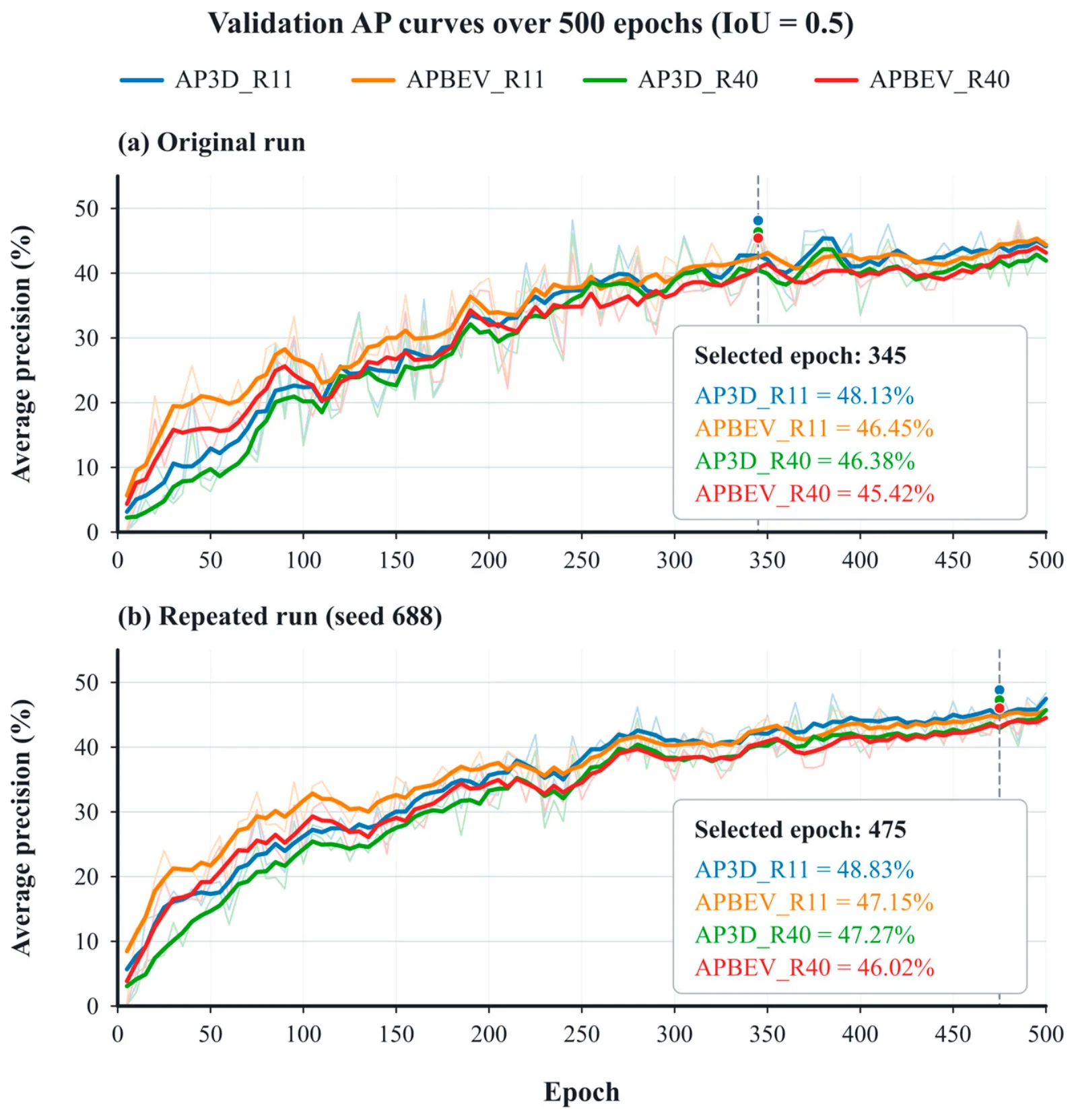
Validation AP curves over 500 epochs at IoU = 0.5 for (**a**) the original training run and (**b**) the repeated run (seed 688). Light-colored lines represent individual validation results, and thick lines show centered five-point moving averages. Dashed vertical lines and markers indicate the checkpoints selected according to *AP*_3*D*,*R*40_ at epochs 345 and 475, respectively. Smoothing is used only for visualization.

Figure 5 compares the validation AP curves of the original training run and the repeated run with seed 688. Light-colored lines show individual validation results, while thick lines show centered five-point moving averages used only for visualization. Both runs exhibit early improvements followed by fluctuations around higher AP levels. According to the checkpoint-selection rule in Section 4.1.3, the checkpoints at epochs 345 and 475 were selected using the unsmoothed *AP*_3*D*,*R*40_ values, respectively.

Across the two runs, *AP*_3*D*,*R*11_, *AP_BEV_*_,*R*11_, *AP*_3*D*,*R*40_, and *AP_BEV_*_,*R*40_ were 48.48 ± 0.49%, 46.80 ± 0.49%, 46.83 ± 0.63%, and 45.72 ± 0.43%, respectively, reported as the mean ± sample standard deviation. The comparable results provide additional evidence of run-to-run consistency under the same experimental settings.

**Figure 6:**
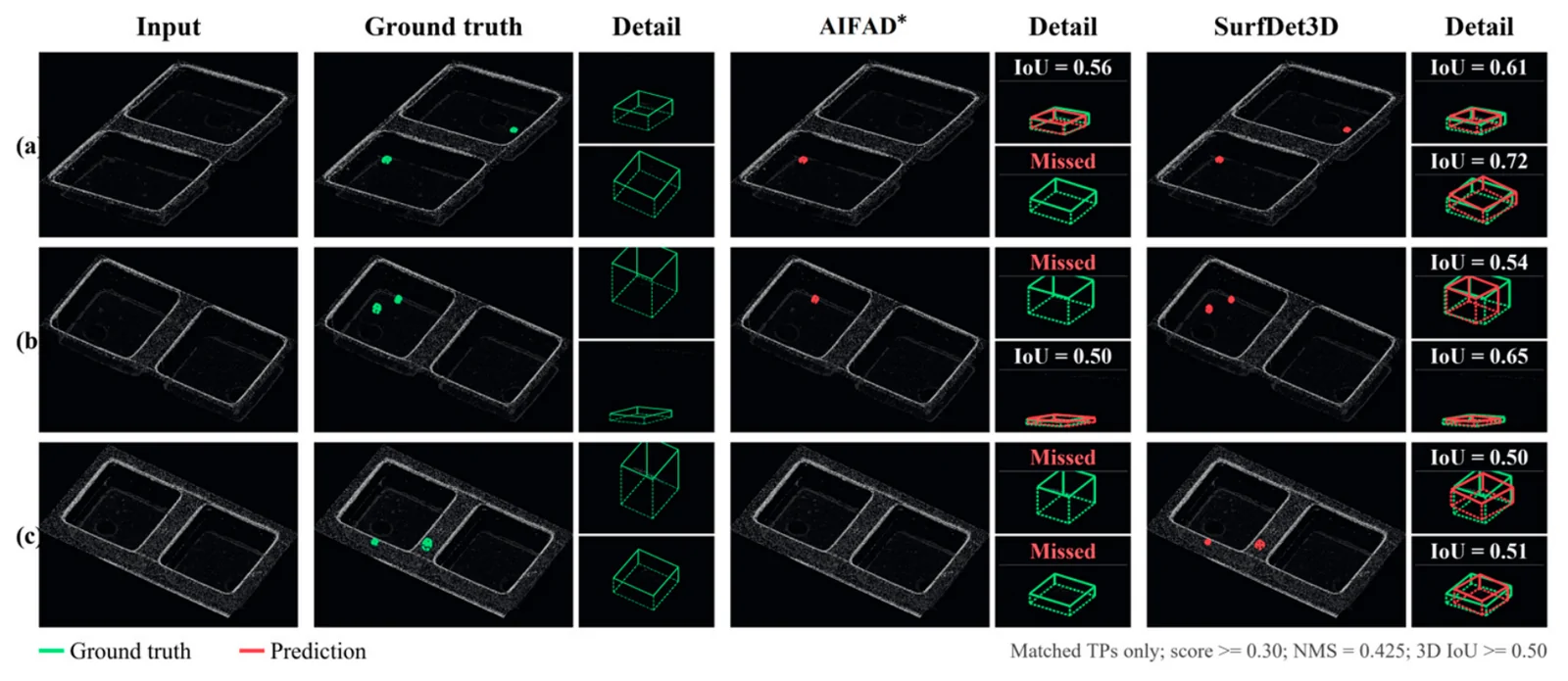
Qualitative comparison of typical detection results between SurfDet3D and AIFAD*: (**a**) Sample o2_h2_l3_r11; (**b**) Sample o3_h4_l4_r2; (**c**) Sample o3_h2_l6_r3.

Figure 6 compares SurfDet3D and AIFAD* on representative samples. In the examples shown, SurfDet3D detects a larger proportion of the small defects and produces bounding boxes that are more closely aligned with the ground-truth centers, dimensions, and orientations. Its predictions also provide more complete spatial coverage for the elongated targets. These observations are consistent with the quantitative improvements reported in Table 1 and illustrate the behavior of SurfDet3D in detecting and localizing small defects with diverse morphologies.

#### 4.4.2. BSSG Feature Response Analysis

**Figure 7:**
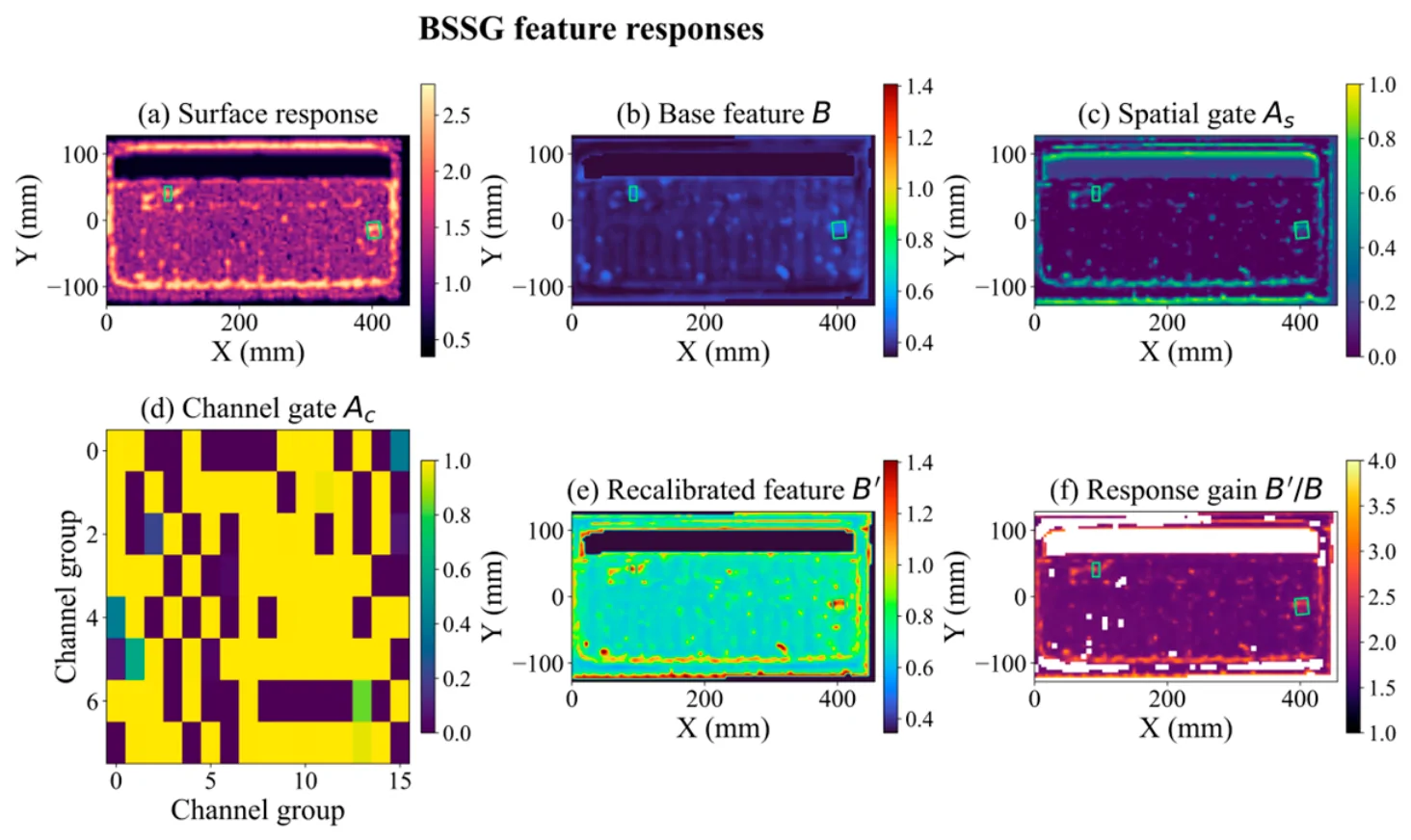
BSSG-guided BEV feature recalibration (sample o1_h1_l1_r2). The rectangular boxes highlight the regions containing defects.

Figure 7 visualizes the recalibration of BEV features by BSSG. The green boxes indicate the BEV projections of the ground-truth defects. Figure 7a–f show the bi-scale surface response, base BEV feature, spatial gate, channel-gate weights, recalibrated feature, and response gain, respectively. For visualization purposes, the 128 channel weights are arranged as a matrix.

Compared with the base features in Figure 7b, the recalibrated features in Figure 7e exhibit stronger and more spatially continuous responses within the defect regions. Figure 7f further presents the relative response gain; locations with near-zero base responses are masked in white to avoid unstable ratios. This visualization illustrates how bi-scale surface statistics guide BEV feature enhancement and is consistent with the BSSG ablation results reported in Table 2.

#### 4.4.3. Localization Quality Fusion Analysis

**Figure 8:**
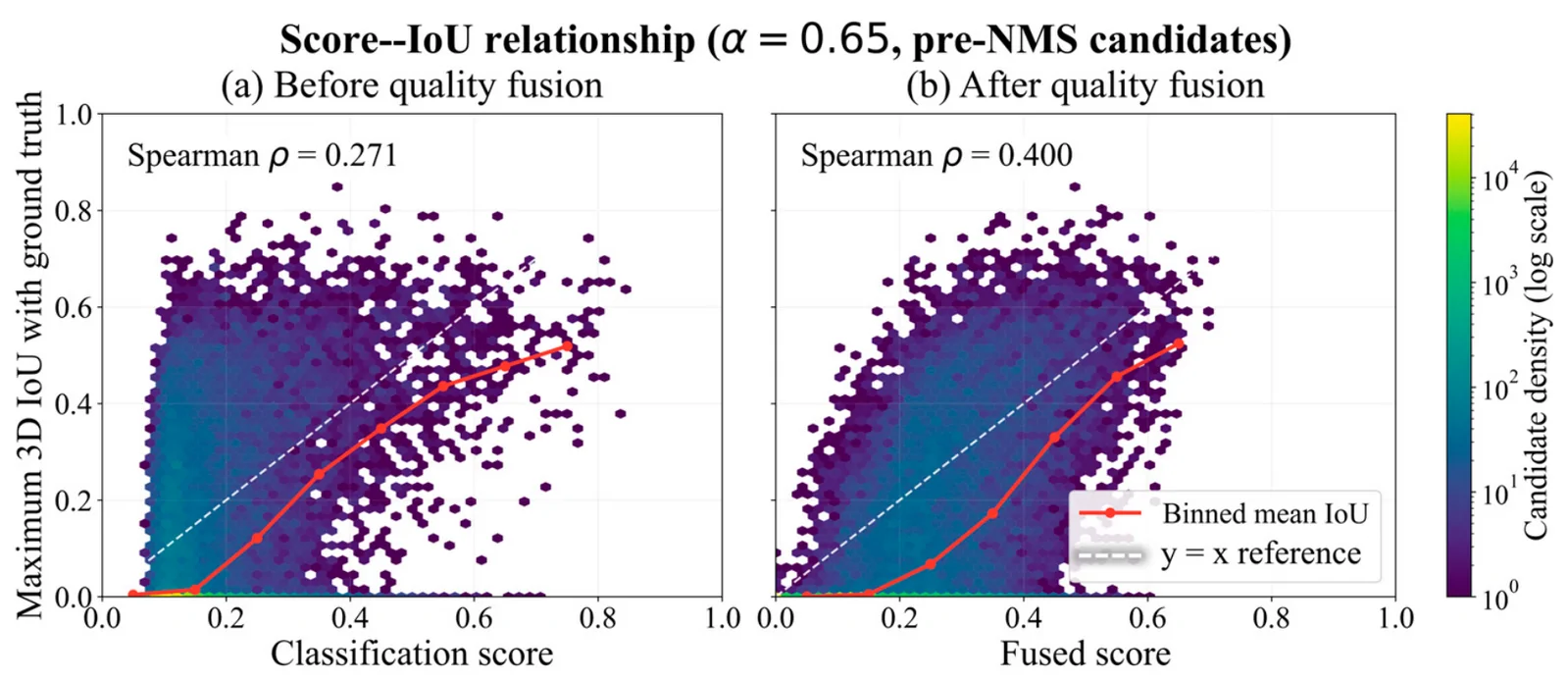
Relationship between candidate scores and maximum rotated 3D IoU before and after localization quality fusion.

Figure 8 analyzes the relationship between candidate scores and maximum rotated 3D IoU before and after localization quality fusion. The analysis includes 152,619 candidate boxes obtained from the 312 validation samples after Top-K selection and classification-threshold filtering but before NMS. For each candidate, the maximum rotated 3D IoU with all ground-truth boxes in the same sample was calculated without one-to-one matching. Of these candidates, 140,454 had a maximum IoU of zero, accounting for approximately 92.0% of the total.

After quality fusion, the Spearman rank correlation coefficient between the candidate scores and rotated 3D IoU increased from 0.271 to 0.400. Because the candidate boxes themselves remained unchanged, this increase indicates stronger consistency between candidate ranking and localization quality over the analyzed candidate set. Together with the AP reductions observed after removing QB in Table 2, this result supports the contribution of localization quality supervision and score fusion to candidate ranking and overall detection performance.

Taken together, the ablation and visualization results show that LRG and BSSG strengthen the representation of subtle local surface geometric anomalies through within-column relative geometry and cross-column surface guidance. JGC and QB improve the localization and candidate ranking of small defects with diverse morphologies through whole-box geometric constraints and localization-quality-aware scoring.

### 4.5. Analysis of Challenging Samples

**Figure 9:**
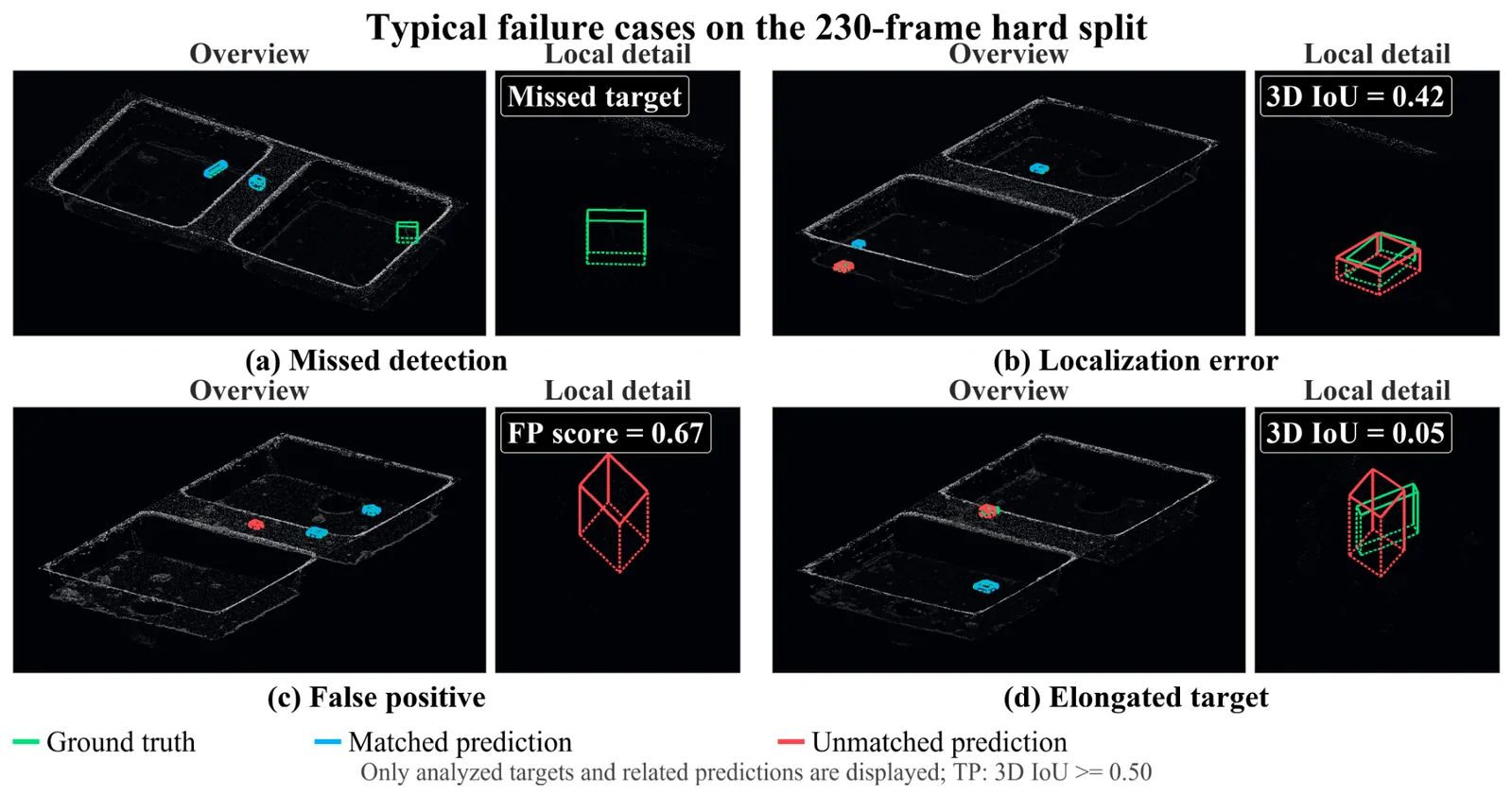
Representative failure cases selected from the 230 challenging defective samples.

Four representative cases were selected from the 230 challenging defective samples excluded from training and checkpoint selection, as shown in Figure 9. The green, blue, and red boxes denote ground-truth boxes, correctly matched predictions, and unmatched predictions, respectively. Only the selected targets and their neighboring predictions are displayed to highlight the failure modes under analysis.

Figure 9a shows a missed target with a weak geometric response, suggesting that sampling, voxel quantization, and feature downsampling may still attenuate subtle anomaly signals. In Figure 9b, the predicted box is spatially close to the ground-truth box but has a 3D IoU of 0.42 and therefore fails to satisfy the matching threshold of 0.5, indicating residual localization error under weak boundary cues. Figure 9c presents a false positive caused by a non-defective local structure, while Figure 9d shows dimension and orientation errors for an elongated target. These localization cases help contextualize the sensitivity to stricter overlap requirements reported in Section 4.2. Further development will target weak-response and elongated defects through higher-resolution geometric encoding and refined boundary and orientation regression, particularly under strict IoU requirements. Deployment-oriented optimization will also balance detection accuracy with the additional feature-processing cost of surface guidance.

## 5. Conclusions

This paper proposes SurfDet3D, a single-stage 3D detector designed to address the reliable representation of subtle local surface geometric anomalies and the accurate localization and reliable candidate ranking of small defects with diverse morphologies. LRG and BSSG strengthen subtle-defect representation through within-column relative geometry and bi-scale cross-column surface guidance, while JGC and QB improve whole-box localization and localization-quality-aware candidate ranking. Under the public CPS3D-Det validation protocol, SurfDet3D achieves *AP*_3*D*,*R*11_, *AP_BEV_*_,*R*11_, *AP*_3*D*,*R*40_, and *AP_BEV_*_,*R*40_ values of 48.13%, 46.45%, 46.38%, and 45.42%, respectively, with only 3.527 M parameters. The ablation results further support the contribution of each proposed component.

Weak-response and elongated defects remain challenging, and detection performance decreases under stricter IoU requirements. The accuracy gains over the local baselines are accompanied by higher inference latency. Future work will explore higher-resolution geometric representations and refined boundary and orientation regression, together with deployment-oriented optimization to improve the accuracy–latency trade-off.

## Figures and Tables

**Table 1:** Detection performance and model complexity on the CPS3D-Det validation set (IoU = 0.5).

	Method	Type	*AP*_3*D*,*R*11_	*AP_BEV_*_,*R*11_	*AP*_3*D*,*R*40_	*AP_BEV_*_,*R*40_	Params
Published benchmark results [15]	Part-A^2^ [16]	Point–Voxel	17.11%	16.24%	16.37%	15.43%	63.79 M
	Part-A^2^-free [16]	Point–Voxel	13.93%	10.91%	14.03%	9.84%	57.00 M
	PV-RCNN [17]	Point–Voxel	24.04%	24.27%	20.40%	21.04%	13.05 M
	PointPillars [18]	Point-based	30.57%	29.36%	28.14%	26.09%	4.81 M
	CenterPoint [19]	Point-based	29.40%	33.40%	28.71%	31.46%	7.76 M
	PillarNet [20]	Voxel-based	35.04%	37.25%	31.37%	34.36%	10.98 M
	SECOND [21]	Voxel-based	34.36%	33.57%	32.90%	31.65%	5.30 M
	SECOND-Multihead [21]	Voxel-based	34.70%	34.99%	32.14%	32.25%	5.58 M
	VoxelNeXt [22]	Voxel-based	40.65%	37.47%	37.77%	34.94%	7.45 M
	AIFAD-2D [15]	Voxel-based	40.67%	39.69%	39.47%	38.41%	4.11 M
	AIFAD [15]	Voxel-based	42.40%	41.13%	40.12%	39.54%	4.19 M
Local experimental results	VoxelNeXt* [22]	Voxel-based	38.52%	33.76%	32.34%	29.13%	7.453 M
	AIFAD* [15]	Voxel-based	40.25%	39.56%	38.11%	37.63%	4.188 M
	SurfDet3D (Ours)	Voxel-based	48.13%	46.45%	46.38%	45.42%	3.527 M

Note: Published baseline results are cited from [15]. Asterisks identify local baseline results. All AP values are percentages evaluated on the CPS3D-Det validation split at IoU = 0.5. Red and blue indicate the best and second-best results, respectively, with higher AP and lower parameter counts preferred.

**Table 2:** Ablation results for the individual components (IoU = 0.5).

Configuration	LRG	BSSG	JGC	QB	*AP*_3*D*,*R*11_	*AP_BEV_*_,*R*11_	*AP*_3*D*,*R*40_	*AP_BEV_*_,*R*40_
Baseline	—	—	—	—	36.82%	34.91%	35.28%	31.88%
w/o LRG	—	√	√	√	43.25%	42.65%	39.13%	41.92%
w/o BSSG	√	—	√	√	43.93%	39.26%	39.46%	38.08%
w/o JGC	√	√	—	√	41.66%	40.57%	40.98%	36.94%
w/o QB	√	√	√	—	38.63%	40.30%	36.79%	35.52%
Full	√	√	√	√	48.13%	46.45%	46.38%	45.42%

Note: “√” and “—” indicate that the corresponding component is enabled and disabled, respectively.

## Data Availability

Publicly available datasets were analyzed in this study. The CPS3D-Det dataset is available at https://github.com/dcsrgh/CPS3D-Det (accessed on 11 October 2026). No new datasets were generated during the current study.

## References

[1] Sun Q., Liu J., Peng Y., Wang A., Wu Z., Chen M. (2020). Effective heat dissipation of high-power LEDs through creation of three-dimensional ceramic substrate with kaolin/graphene suspension. J. Alloys Compd..

[2] Monti R., Coppola F., Gasbarri P., Lecci U. (2010). Residual stress brazing process induced in hybrid package for ISP applications. Acta Astronaut..

[3] Yang Y., Dorn-Gomba L., Rodriguez R., Mak C., Emadi A. (2020). Automotive power module packaging: Current status and future trends. IEEE Access.

[4] Teng L., Yu Z., Zhu D., Hao C., Jiang N. (2024). Antenna array design based on low-temperature co-fired ceramics. Micromachines.

[5] Huang Y.-P., Su C.-M., Basanta H., Tsai Y.-L. (2021). Imbalance modelling for defect detection in ceramic substrate by using convolutional neural network. Processes.

[6] Liao D., Cui Z., Liao X., Zheng Q., Wu N. (2023). A lightweight convolutional neural network for recognition and classification for Si3N4 chip substrate surface defects. Ceram. Int..

[7] Xin K., Chen G., Fei Z., Chen J., Qin X., Ding X. Semantic segmentation based defect detection algorithm for ceramic substrates. Proceedings of the China Automation Congress (CAC).

[8] He Y., Cai C., Chen G., Hu J., Hu S., Fu J. (2025). PFEI-Net: A profound feature exploration and interaction network for ceramic substrate surface defect detection. Expert Syst. Appl..

[9] Li H., Yu R., Guo B. (2026). Dynamic cross characterization network for few-shot IC package substrates surface defect segmentation. IEEE Trans. Ind. Inform..

[10] Guo B., Wang Y., Yu R. (2026). EDNet: Zero-shot classification for ceramic package substrates surface defect with embedding diffusion network. Pattern Recognit..

[11] Li H., Yu R., Guo B., Zhen S., Zhang Z. (2026). Hybrid-space interaction network for single-image super-resolution in industrial intelligent detection. IEEE/ASME Trans. Mechatron..

[12] Huang C.-Y., Lin I.-C., Liu Y.-L. (2022). Applying deep learning to construct a defect detection system for ceramic substrates. Appl. Sci..

[13] Wang Y., Sun L., An B., Yu R. (2026). MulPViT-SimAM: An electronic substrate defect detection framework for addressing class imbalance problems. Machines.

[14] Guo B., Zuo Q., Yu R. (2025). Point cloud segmentation of integrated circuits package substrates surface defects using causal inference: Dataset construction and methodology. arXiv.

[15] Yu R., Zhao Z., Zhen S. (2026). Auxiliary information flow for 3D industrial defect detection on IC ceramic package substrate surfaces: Dataset and benchmark. IEEE Trans. Circuits Syst. Video Technol..

[16] Shi S.S., Wang Z., Shi J., Wang X., Li H. (2021). From Points to Parts: 3D Object Detection From Point Cloud With Part-Aware and Part-Aggregation Network. IEEE Trans. Pattern Anal. Mach. Intell..

[17] Shi S.S., Guo C., Jiang L., Wang Z., Shi J., Wang X., Li H. PV-RCNN: Point-Voxel Feature Set Abstraction for 3D Object Detection. Proceedings of the IEEE/CVF Conference on Computer Vision and Pattern Recognition (CVPR), IEEE Conference on Computer Vision and Pattern Recognition.

[18] Lang A.H., Vora S., Caesar H., Zhou L., Yang J., Beijbom O. PointPillars: Fast Encoders for Object Detection from Point Clouds. Proceedings of the 32nd IEEE/CVF Conference on Computer Vision and Pattern Recognition (CVPR), IEEE Conference on Computer Vision and Pattern Recognition.

[19] Yin T.W., Zhou X., Krahenbuhl P. Center-based 3D Object Detection and Tracking. Proceedings of the IEEE/CVF Conference on Computer Vision and Pattern Recognition (CVPR), IEEE Conference on Computer Vision and Pattern Recognition.

[20] Shi G.S., Li R., Ma C. PillarNet: Real-Time and High-Performance Pillar-Based 3D Object Detection. Proceedings of the 17th European Conference on Computer Vision (ECCV), Lecture Notes in Computer Science.

[21] Yan Y., Mao Y., Li B. (2018). SECOND: Sparsely embedded convolutional detection. Sensors.

[22] Chen Y.K., Liu J., Zhang X., Qi X., Jia J. VoxelNeXt: Fully Sparse VoxelNet for 3D Object Detection and Tracking. Proceedings of the IEEE/CVF Conference on Computer Vision and Pattern Recognition (CVPR), IEEE Conference on Computer Vision and Pattern Recognition.

[23] Woo S., Park J., Lee J.-Y., Kweon I.S. CBAM: Convolutional block attention module. Proceedings of the European Conference on Computer Vision (ECCV).

[24] Zheng Z., Wang P., Liu W., Li J., Ye R., Ren D. (2020). Distance-IoU loss: Faster and better learning for bounding box regression. Proceedings of the AAAI Conference on Artificial Intelligence.

